# Integration of physio-biochemical, biological and molecular approaches to improve heavy metal tolerance in plants

**DOI:** 10.1007/s13205-025-04248-y

**Published:** 2025-03-06

**Authors:** Swathi Shivappa, K. P. Amritha, Siddharth Nayak, Harsha K. Chandrashekar, Sachin Ashok Thorat, Arya Kaniyassery, Nisha Govender, Muthu Thiruvengadam, Annamalai Muthusamy

**Affiliations:** 1 Department of Plant Sciences, Manipal School of Life Sciences, Manipal Academy of Higher Education (MAHE), Manipal, Karnataka 576104 India; 2 Institute of Systems Biology (INBIOSIS), Universiti Kebangsaan Malaysia UKM, 43600 Bangi, Selangor Malaysia; 3 Department of Applied Bioscience, College of Life and Environmental Sciences, Konkuk University, Seoul, 05029 South Korea

**Keywords:** Heavy metal, Strategies, Bioremediation, Phytoextraction, Metal tolerance

## Abstract

Heavy metal toxicity hinders plant growth and development by inducing oxidative stress, decreasing biomass, impairing photosynthesis, and potentially leading to plant death. The inherent defense mechanisms employed by plants, including metal sequestration into vacuoles, phytochelation, cell wall metal adsorption and an enhanced antioxidant system can be improved via various approaches to mitigate heavy metal toxicity. This review primarily outlines plants direct and indirect responses to HM stress and the tolerance mechanisms by which plants combat the toxic effects of metals and metalloids to understand the effective management of HMs and metalloids in the soil system. Furthermore, this review highlights measures to mitigate metal and metalloid toxicity and improve metal tolerance through various physio-biochemical, biological, and molecular approaches. This review also provides a comprehensive account of all the mitigative approaches by comparing physio-biochemical, biological and molecular approaches. Finally, we compared all the mitigative approaches used in monocotyledonous and dicotyledonous to increase their metal tolerance. Although many studies have compared monocot and dicot plants based on metal toxicity and tolerance effects, comparisons of these mitigative approaches have not been explored.

## Introduction

Heavy metals (HMs) are a class of metals and metalloids that are toxic even at minute levels (parts per billion) because of their relatively high density for human health and the environment (Leong and Chang 2020). Prolonged exposure to HM may threaten wildlife populations and communities, and consequently pose a risk to biodiversity (Tovar-Sánchez et al. 2018). Zinc (Zn), gold (Ag), copper (Cu), iron (Fe), chromium (Cr), nickel (Ni), palladium (Pd), platinum (Pt), lead (Pb), arsenic (As), mercury (Hg), and cadmium (Cd) are a few examples (Leong and Chang 2020). The increasing accumulation of HMs in agricultural fields is a rising concern across the globe because of its immediate influence on human health (Kaur et al. 2021). Although human activity and industrial development have significantly increased living standards, they have also led to environmental issues such as the accumulation of HMs in water and soil resources (Ali et al. 2019). Some metals/metalloids, such as Fe, Cu, manganese (Mn), molybdenum (Mo), selenium (Se), Zn, and boron (B), are required for the proper functioning of plants, whereas others including Pb, Hg, As, Cd, and Cr are considered non-essential and sometimes hazardous for plants (Kaur et al. 2021). Currently, HM pollution is still one of the most significant environmental issues and has garnered much attention (Wu et al. 2019).

Both natural processes and anthropogenic activities lead to the accumulation of transition elements such as Fe, Co, Ni, Cu, and Zn, which exist in soil and water at potentially hazardous concentrations. Volcanic activity, weathering of rocks and minerals, and air deposition are some natural sources of metals in the soil. Industrial processes such as mining and smelting and agricultural practices such as the application of pesticides and fertilizers are some of the anthropogenic sources of metals in soil (Zhang et al. 2023a, b). Owing to their unalterable nature and lasting persistence in the environment, metals/metalloids are pollutants of concern, and trace metal contamination is a problem that is becoming a growing issue worldwide (Lado et al. 2008). These metals are systemic toxicants that have been linked to several harmful health issues in people, such as diabetes, hearing loss, developmental abnormalities, cardiovascular disease, immunologic and neurologic disorders, and different types of cancer (Tchounwou et al. 2012; Mitra et al. 2022). In recent decades, the unanticipated negative impact of metal contaminants on crop quality has compromised human health and food security. Certain HMs, such as Cu, Fe, Zn, and Cr(III), are crucial components of metabolic processes, such as enzyme cofactors and cytochromes that are essential for living organisms. However, when these metals accumulate at relatively high levels, they can pose serious health risks to humans. Metalloids and HM can interfere with the body’s metabolic functions, leading to illnesses and, in extreme cases, even death (Rai et al. 2019).

The area where plant roots and soil interact is known as the rhizosphere. It serves as the first line of defense for plants against pollutants and the entry point for nutrients and microorganisms to act on plant roots and enter the plant through the root system to participate in material circulation (Wang et al. 2022). Since plants are sessile organisms, the changing climate exposes them to various biotic and abiotic stresses that negatively impact their growth and development (Sarris and Christodoulakis 2024). Toxic metals/metalloids hinder the function of plants primarily by displacing ions and vital biomolecules from their binding locations and/or by competing with essential metal ions for uptake at the plant’s absorption level at the root surface. Thus, HMs denature crucial functional groups, making them inactive and generating reactive oxygen species (ROS), which interact with biomolecules, deteriorate them, and interfere with metabolic processes (Ghori et al. 2019). Metal/metalloids enter plant roots through phosphate transporters under anaerobic conditions, e.g., arsenate (As(V)). In contrast, arsenite (As(III)) and methylated As(III) are primarily absorbed through aquaporin channels and accumulate in plants (Farooq et al. 2016). When exposed to HMs, plants suffer greatly from oxidative stress, which damages and alters cellular homeostasis, ultimately resulting in plant death (Emamverdian et al. 2022a, b).

The spatial distribution of toxic metals/metalloids in plants is influenced by plant growth, changes in bioavailability caused by the immobilization of soil pollutants, and changes in element absorption ability (Tan et al. 2023). One of the main strategies for mitigating metal toxicity is the production of phytocompounds, which are composed of low-molecular-weight compounds such as phenolics, proline, salicylic acid, phytochelatins, and metallothioneins, as well as high-molecular-weight antioxidant enzymes (Rizvi et al. 2023). Plants have developed several signaling mechanisms for HM toxicity, such as the incorporation of protein molecules, ROS, calcium ions, nitric oxide (NO) and hormones. Under highly toxic conditions, plants require external help to cope with toxic effects. Several techniques such as phytoremediation, i.e., the use of plants to remove pollutants and exogenous applications of nutrients, organic amendments, and growth regulators, protect plants from the harmful effects of HMs by strengthening their defense mechanisms and enhancing their growth (Ali and Gill 2022).

A greater understanding of the physio-biochemical response and mechanisms underlying soil‒plant metal/metalloid transfer is necessary before developing effective remediation solutions. This review briefly examines the impact of metals and metalloids on plant physiology and biochemical aspects. Although many research papers and reviews have mentioned the role of various mitigative approaches for heavy metal toxicity in plants, a comprehensive review of all physio-biochemical, biological and molecular approaches is lacking. Thus, the present study shed light on the physio-biochemical, bioremediation and molecular strategies as viable ways to reduce the negative effects of metal and metalloid toxicity on plants. Even though numerous studies have compared the effects of metal toxicity and tolerance in monocot and dicot plants, a comparative analysis of these plants’ responses to mitigative approaches is yet to be explored. Thus, our review aimed to do a comparative study of these physio-biochemical, biological and molecular approaches in monocot and dicot plants.

## Plant responses to metal/metalloid toxicity

**Fig. 1:**
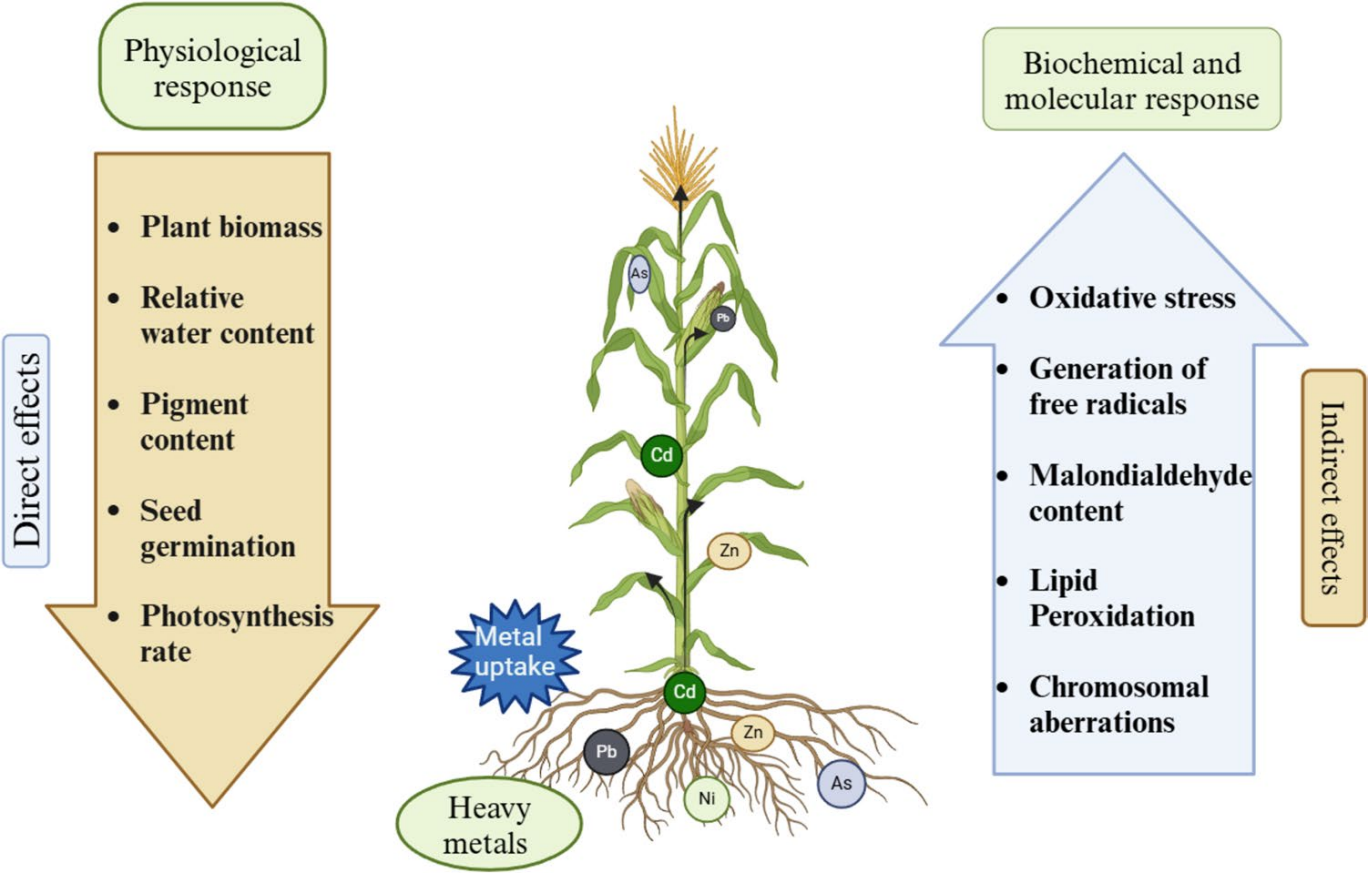
Plant response to toxic metal/metalloids in soil: illustrates the physio-morphological, biochemical, and molecular responses exhibited by plants due to metal toxicity. (*As* Arsenic, *Cd* Cadmium, *Ni* Nickel, *Pb* Lead, *Zn* Zinc). Image modified from (Raza et al. 2022). Created using BioRender.com

The development of strategies to lessen the negative effects of metal/metalloid toxicity on plants require an understanding of how plants react to these substances. Table 1 lists the various physiological, biochemical, and molecular responses exhibited by monocot and dicot plants when they are exposed to toxic metals or metalloids. Figure 1 illustrates the direct and indirect effects of metal and metalloid toxicity.

**Table 1:** Metal toxicity-induced physiological and biochemical responses in monocot and dicot plants

Crop	Metal/metalloid	Response	References
Monocot			
*Hordeum vulgare*	Boron	Upregulation of chitin-binding lectin precursor, ubiquitin carboxyl-terminal hydrolase, and serine/threonine-protein kinase *AFC2* genes. Activated Ca^2+^ calmodulin system	Tombuloglu et al. (2015)
*Hordeum vulgare*	Arsenic	Inhibition of plant growth, impaired morphology of bark layer cells	Burachevskaya et al. (2021)
*Oryza sativa*	Antimony, selenium	Increased MDA content, reduced biomass, reduced soluble protein contents	Feng et al. (2016)
*Oryza sativa*	Nickel	Increased oxidative damage and osmotic stress, altering redox balance and MGO accumulation resulting in growth retardation	Hasanuzzaman et al. (2019)
*Oryza sativa*	Antimony	Reduced shoot and root biomass, leaf water moisture content, water use efficiency, stomatal conductance, net photosynthetic rate, and transpiration rate. Enhanced synthesis of glycoconjugates	Zhu et al. (2022)
*Triticum aestivum*	Cadmium	Reduced nutrient uptake, photosynthetic pigments, antioxidants, and biomass production	Hussaan et al. (2023)
Dicot			
*Allium cepa*	Chromium, arsenic	Decreased root length, and protein content	Gupta et al. (2018)
*Brassica juncea*	Arsenic, chromium, copper	Inhibits growth and productivity	Ahmad et al. (2020)
*Brassica napus*	Chromium	Increased ROS and oxidative damage	Zaheer et al. (2020)
*Citrus sinensis*	Aluminum	Increased oxidative stress, membrane damage, root damage, and injury	Riaz et al. (2018)
*Dahlia pinnata*	Arsenic	Reduced chlorophyll and carotenoid contents. Activities of antioxidant enzymes such as GST, APX, CAT, POD, and SOD were affected	Raza et al. (2019)
*Helianthus annuus*	Cadmium	Reduced biomass, carotenoid, and chlorophyll concentrations	Saidi et al. (2014)
*Raphanus sativus*, *Brassica napus*	Antimony	Reduced seed germination and reduced root elongation	Liang et al. (2018)
*Solanum nigrum*	Lead, arsenic, cadmium	Inhibition of plant length, metal detoxification	Li et al. (2020)
*Spinacia oleracea*	Lead, arsenic	Reduced plant biomass and pigment content	Zubair et al. (2019)
*Spinacia oleracea*	Lead, arsenic	Decreased plant biomass and pigment contents and provoked oxidative stress by increased H_2_O_2_ production in roots	Natasha et al. (2022)
*Spinacia oleracea*	Chromium	Reduced contents of anthocyanin, carotenoid, and total chlorophyll	Ali et al. (2023)
*Trachyspermum ammi*	Arsenic	Enhanced ROS production, exudation of organic acids, and increased oxidative stress	Sun et al. (2022)
*Trifolium pratense*	Lead	Altered levels of antioxidant enzymes, MDA, proline, soluble substances, and photosynthetic pigments	Meng et al. (2022)
*Vigna radiata*	Copper	Declined activities of antioxidants GSH, APX, and CAT, and increased oxidative stress and cell death	Gaur et al. (2021)

*Ca* Calcium; *MDA* malondialdehyde; *MGO* methylglyoxal; *ROS* reactive oxygen species; *GST* glutathione S-transferase; *APX*, ascorbate peroxidase; *CAT* catalase; *POD* guaiacol peroxidase; *SOD* superoxide dismutase; *H*_*2*_*O*_*2*_ hydrogen peroxide; *GSH* glutathione

## Physiological response

HMs and metalloids like As exhibit toxic effects primarily through decreased plant biomass, growth, yield (Mousavi et al. 2020; Kaya and Ashraf 2022; Sun et al. 2022) and chlorophyll levels (Zulfiqar et al. 2023a, b). HMs decrease fresh and dry leaf weights and reduce photosynthetic pigments (Gharib and Ahmed 2023). In terms of biomass and plant growth, As(III) is considered more toxic than As(V) under both acute and chronic exposure conditions (Pandey et al. 2023). The concentrations of carotenoids and chlorophylls decreased at relatively high As levels in *Dahlia pinnata* plants (Raza et al. 2019). Toxicity impairs the expression of potential genes involved in photosynthesis, mitochondrial electron transport, and lipid biosynthesis metabolism (Hwang et al. 2017). Pb and As are two extremely hazardous and carcinogenic metals/metalloids that affect plant biomass and pigment contents in spinach (Zubair et al. 2019). Sunflower plants exposed to Cd produced less biomass, had lower concentrations of carotenoids and chlorophyll, and exhibited markedly increased Cd accumulation in both the roots and the shoots (Saidi et al. 2014). Nutrient uptake, pigment content, antioxidant content, biomass production, and plant growth are reduced by increased concentrations of Cd (Hussaan et al. 2023). Although B is a necessary plant micronutrient, excess B in the soil can be toxic to plants and reduce their yield (Tombuloglu et al. 2015). Antimony (Sb) (≥ 10 mg/L) inhibited the germination of radish and rape seeds (Liang et al. 2018). Sb has a negative effect on photosynthesis and leaf fluorescence at high concentrations (Abbasi et al. 2022). Cr, a highly mobile HM, is considered very harmful to plants because it slows plant growth and biomass production (Alam et al. 2021) and impacts the composition and gas exchange parameters of plants (Zaheer et al. 2020). A reduction in root growth and root hair surface area and increased root death are the primary responses of toxicity, as roots are the first plant parts that are exposed to HM at the soil‒plant interphase. The thickening of cell walls caused by metal toxicity increases apoplast resistance to water flow. This reduces the flow of water into the vascular system, reducing the exudation of root sap and causing a water deficit in the leaves (Rucińska-Sobkowiak 2016). The reproductive potential of plants is also negatively affected (Bhalla and Garg 2021).

## Biochemical response and molecular response

Toxic metals and metalloids typically negatively impact biochemical processes in plants by obstructing functional groups or displacing essential ions (Ghori et al. 2019). The induction of oxidative stress in response to metal/metalloid toxicity is one of the primary responses (Mousavi et al. 2020). Exposure to As altered the activities of antioxidant enzymes, including glutathione S-transferase (GST), ascorbate peroxidase (APX), catalase (CAT), guaiacol peroxidase (POD), and superoxide dismutase (SOD) (Raza et al. 2019). Increased expression of the oxidative stress markers malondialdehyde (MDA), methylglyoxal (MGO), and hydrogen peroxide (H_2_O_2_) and increased electrolyte leakage have been observed under As stress (Zulfiqar et al. 2023a, b) and Cr stress (Alam et al. 2021). Excessive Cr increases ROS and causes oxidative damage in *Brassica napus* roots and leaves (Zaheer et al. 2020). Cu toxicity in mung bean seedlings also caused oxidative stress and cell death. A decrease in antioxidant, glutathione (GSH), APX, and CAT activity was observed, resulting in decreased viability (Gaur et al. 2021). Pb reduces plant matter and vitamin C contents while increasing the activities of antioxidant enzymes, flavonoids, and MDA (Fatemi et al. 2021). Under conditions of excess Pb, oxidative stress is triggered by increased production of H_2_O_2_ in the roots of spinach plants. Furthermore, under metal/metalloid stress, the antioxidant system, which includes SOD, CAT, POD, and APX, is activated (Zubair et al. 2019). Rice plants exposed to Ni toxicity suffer from oxidative damage and osmotic stress, altering redox balance and antioxidant defense. As a result, MGO accumulates, resulting in further harm and growth retardation (Hasanuzzaman et al. 2019). The B-excess stress response of barley is regulated primarily by genes related to the stress response, cell wall, membrane integrity and cytoskeleton formation, Ca^2+^/calmodulin framework, phospholipase activity, and signal transduction (Tombuloglu et al. 2015). Al hyperaccumulation may lead to oxidative stress, membrane damage, root damage, and injury in orange plants (Riaz et al. 2018). As induces a toxic response through increased production of ROS, exudation of organic acids, and increased oxidative stress in *Trachyspermum ammi* seedlings. In addition, it disturbs plant nutritional balance and ultrastructure of plants (Sun et al. 2022).

Cd toxicity considerably reduced the mitotic index and progressively increased the rate of chromosomal aberrations. As and Cd toxicity increased chromosomal aberrations in the root meristem and decreased the mitotic index, indicating that these compounds are carcinogenic and mutagenic agents (Gupta et al. 2018). The accumulation of As in *Hordeum sativum* tissues has a negative effect on plant morphological and ultrastructural characteristics. Metal toxicity was observed to cause detrimental alterations in the cells of the root bark layer as well as a decrease in the size of the chlorophyllic parenchyma in the leaves (Burachevskaya et al. 2021). Al exposure changed the phloem and mesophyll cell framework, decreased the number of stomata and root hairs, lengthened the leaf epidermal cells and root hairs, and decreased the contents of certain minerals (Singh et al. 2011).

At specific concentrations, metal toxicity can stimulate the production of GSH and related enzymes and other S-rich compounds, which help plants survive when exposed to metal/metalloid toxicity (Ahmad et al. 2020). Under both Cd and As exposure, the soybean plants presented a noticeably high homophytochelatin (hPC) content, which contributed to the thiol pool in the shoots. The involvement of hPCs in the As detoxification process appears more evident, indicating lower growth inhibition of roots in response to higher thiol concentrations (Vázquez et al. 2009). Metal/metalloid-chelating amino acid synthesis, PC production, and PC-metal/metalloid complex sequestration in vacuoles are the mechanisms for metal detoxification. As-PC complexes need to be enclosed in vacuoles to fully detoxify As, whereas Cd detoxification can occur simply through PC–Cd binding. Moreover, PCs may combine vital nutrients to form complexes during HM stress. Barley contains a vacuolar PC-Cd transporter for eliminating Cd (Song et al. 2014). The analysis of PC synthesis in plants under Cd and Hg stress showed that Cd was the most effective inducer of PCs. While Cd and Hg tend to synthesize PCs, As is more likely to synthesize small thiols such as GSH and γ-Glu-Cys (Dago et al. 2014).

Although both monocots and dicots are effected by toxic heavy metals, one study showed that the applied metal dose had a greater detrimental effect on maize root growth, whereas soybean plants exhibited more pronounced defense responses and were generally more tolerant to the metal doses tested than maize plants (Piršelová et al. 2011).

## Approaches to enhance heavy metal and metalloid tolerance in plants

**Fig. 2:**
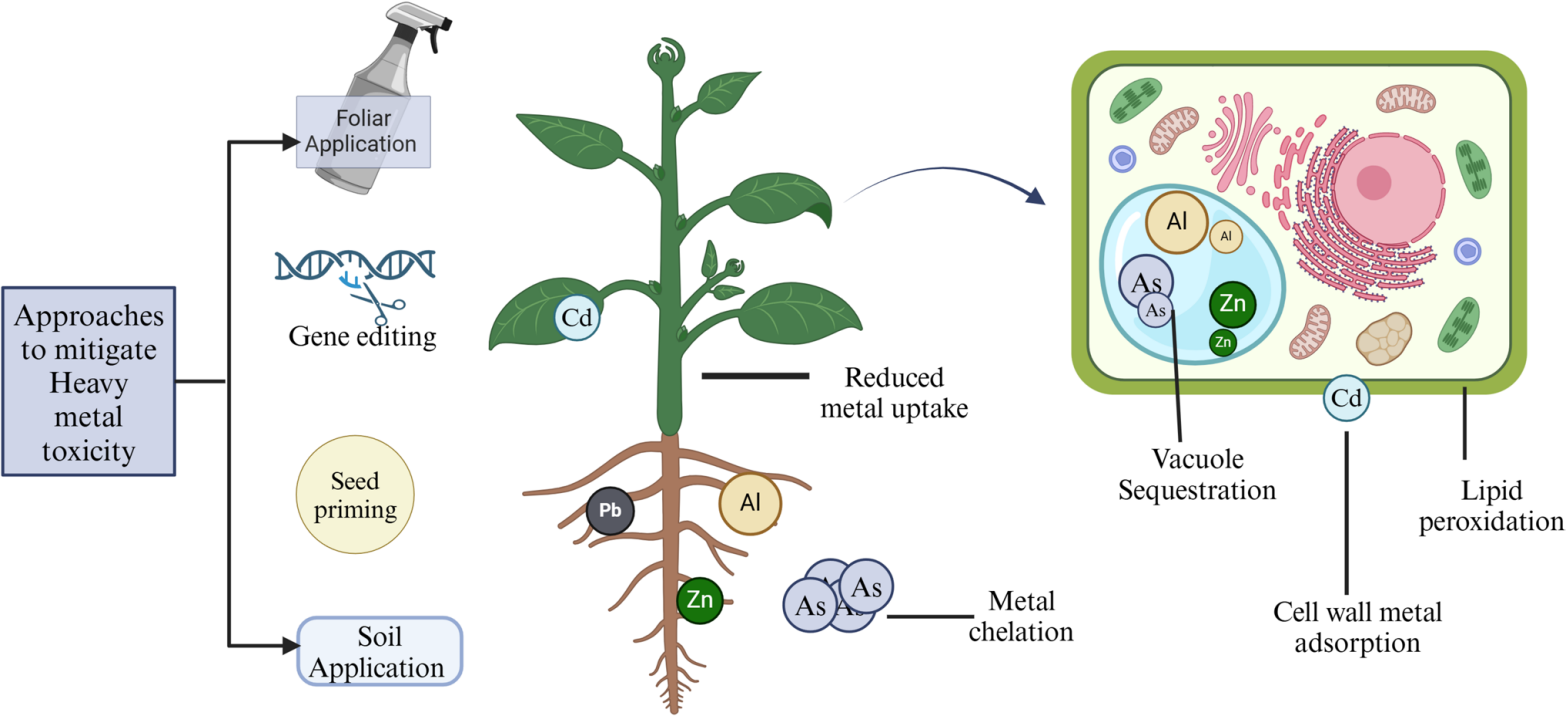
Techniques for enhancing plant metal tolerance: illustrates the different defense mechanisms plants use against metals and metalloid toxicity, which can be enhanced by a variety of physiochemical, biological, and molecular techniques. (*Al* Aluminum, *As* Arsenic, *Cd* Cadmium, *Ni* Nickel, *Pb* Lead, *Zn*—Zinc). Created using BioRender.com

In this review, we have outlined the several approaches to mitigate heavy metal toxicity and the plants response to the same (Fig. 2).

### Inorganic supplements

#### Silicon (Si)

Si, a quasiessential element (Ur Rahman et al. 2021), is crucial for enhanced plant development, yield, photosynthetic characteristics (Shetty et al. 2021), plant biomass, chlorophyll content, root morphology, and enzyme activity during HM stress, which aid in plant tolerance by the development of HM precipitation with Si (Wang et al. 2023a, b). In many plants, Si reduces toxic metal uptake and root-to-shoot translocation (Vieira Filho and Monteiro 2020). Under HM stress, Si application can improve defense mechanisms, cell damage repair, cell homeostasis, and metabolism regulation (Khan et al. 2021). Si reduced the MDA and H_2_O_2_ concentrations and increased the SOD and POD activities in wheat leaves, thereby mitigating the effects of oxidative stress. Si can reduce Cd toxicity in wheat plants by decreasing lipid peroxidation, increasing plant growth, and enhancing antioxidant capacity (Shi et al. 2018). Si induces biomineralization, i.e., the formation of nanostructures on the root surface through cell-induced self-assembly in plant roots, increasing mechanical strength and reducing plant abiotic and biotic stress (Feng et al. 2023). Supplementation with Si increased the total chlorophyll content, translocation factor, relative moisture content, and gas exchange parameters of the leaves of Cr-stressed tomato plants. Si partially reversed Cr toxicity by adjusting the levels of osmoprotectants, the functions of antioxidant enzymes, the glyoxalase system, and the Asc-Glu cycle (Alam et al. 2021). Si predominantly stimulates root lignification, which is most likely the cause of decreased Sb absorption and translocation to the shoot (Shetty et al. 2021). Si supplementation, along with NO induction, mitigated the Cu toxicity of mung bean plants by limiting the accumulation of oxidative stress biomarkers and enhancing the activity of antioxidant enzymes (Gaur et al. 2021). The harmful effects of Al can be reduced by the addition of Si, which strengthens the antioxidant system of plants, improves physiological and morphological characteristics such as leaf architecture, and facilitates better absorption of light, which increases the liquid assimilation rate and increases the immediate carboxylation efficiency of Rubisco (Aguilar et al. 2023). Exogenous Si addition preserved the leaf and root structures and the mineral composition of Zn and Mg that were previously depleted by excess Al in rice plants (Singh et al. 2011). Si enhances *Solanum nigrum*’s ability to withstand Cd by reducing Cd uptake in roots, dispersing Cd throughout growing and aging leaves, and reducing oxidative stress caused by Cd in plants (Liu et al. 2013). Si promoted the formation of an apoplastic barrier in the roots and increased the amount of Mn plaque on the root surface, contributing to Cd tolerance in *Avicennia marina* plants. Si considerably reduced the absorption of Cd and decreased the growth inhibition caused by Cd stress, which may be due to the effect of Si on the root anatomy (Zhang et al. 2013). Si application decreased the phytotoxicity of excess Zn and Cd in rice plants, through root system symplastic pathways, rice plants treated with Si experienced a reduction in Cd accumulation throughout all three growth phases and increased photosynthetic activity and antioxidant enzymes (Huang et al. 2018). The antioxidant systems CAT, SOD, dehydroascorbate reductase, GR, and GST are activated in response to either hydrosulfite (H_2_S) or Si supplied alone or in combination. Combined supplementation of both Si and H_2_S had a greater effect on increasing growth and important metabolic processes, as well as lowering the tissue As content in tomato plants (Kaya and Ashraf 2022). Foliar application of silica sol along with soil application of iron-modified biochar enhances plant yield and productivity by lowering As and Cd bioavailability in soil as well as in rice grain (Pan et al. 2019). The combined application of Si and sodium nitroprusside (SNP) reduces Cr stress in *Brassica juncea* by enhancing the morphology, physiology, biochemistry, and molecular characteristics of the plants through increased antioxidant activity and ROS and MDA detoxification (Sharma et al. 2023). Si in calcium silicate increased the malate/oxalate ratio, citrate synthase activity, ascorbate/glutathione ratio, and phytohormone content and aided in mitigating Zn toxicity through metal sequestration by precipitation with Si. In addition, it reduces ROS levels and HM root-to-shoot translocation and minimizes oxidative stress in plants (Paradisone et al. 2022). Adding Si to wheat crops dismantled the harmful effects of Cd on wheat leaves by increasing the dry biomass, leaf area, and total chlorophyll content. Plants are protected from wilting and flaccid cells by Si incorporation, which significantly increases the relative water content and enhances membrane rigidity (Ur Rahman et al. 2021). The accumulation of Cd and Pb in tobacco plants was more successfully reduced by mineral Si fertilizers than by organic Si fertilizers. Although the photosynthetic parameters and chlorophyll content increase more quickly with mineral Si fertilizer, the POD and APX contents increase more quickly with organic Si fertilizers (Wang et al. 2023a, b).

#### Boron (B)

B fertilizers increase rhizophore pH and stabilize the plant cell wall thus contributing to reduced metal uptake (Yang et al. 2022). Administering B fertilizer to wheat can decrease Cd uptake by regulating the expression of genes linked to antioxidant defense and Cd transport and detoxification; these genes include *TRIAE5660* and *TRIAE5770*, which inhibit Cd absorption; *TCONS1113* and *TRIAE5370*, which detoxify Cd into the vacuole; and *TRIAE5770* and *TRIAE1060*, which transport Cd to grains or spikes. The metabolic expression of metabolites involved in Cd detoxification and stress-related pathways is altered (Qin et al. 2022). Rice growth under Cd toxicity can be increased by B supplementation, which activates the antioxidant defense mechanism in roots and improves Cd adsorption on cell walls. The addition of B increased the root length, volume, and surface area and increased the total chlorophyll content of the plants thereby facilitating the mitigation of the adverse impacts of Cd toxicity (Riaz et al. 2021a, b). Boron supply to Cd-stressed plants enhances cell wall formation, which in turn acts as the primary barrier for Cd uptake by plants by providing Cd binding sites through pectin, cellulose, and hemicellulose components (Riaz et al. 2021a, b). In addition, B greatly decreased Cd influx, movement, and storage in rice seedlings. Increased lignin and pectin production as well as the demethylation of pectin allow for Cd adsorption in root cell walls, which decreases the number of cytosolic free Cd ions, thereby decreasing Cd toxicity. Second, it downregulated the expression of numerous Cd-induced transporter-related genes, such as *heavy metal ATPase2* (*HMA2)* and *natural resistance-associated macrophage protein1* (*Nramp1*) (Huang et al. 2021). B application helps mitigate Cd stress through Cd detoxification into vacuoles and reduces the uptake of Cd in shoots at the elongation stage through the hyperaccumulation of Cd in roots (Qin et al. 2022). Supplying B to plants that are stressed by Al promotes root development and improves photosynthesis while lowering oxidative stress. By limiting entrance into the symplast, the B supply also decreases the amount of Al delivered to other plant regions. In addition, it facilitates the maintenance of protein integrity and controls the actions of proline, secondary metabolites, and antioxidant enzymes (Riaz et al. 2018). Al toxicity increases when plants are B deficient, considering that more unmethylated pectin provides more binding sites for Al in the cell wall (Stass et al. 2007). Thus, the application of B fertilizer can be a valuable strategy for lowering the toxicity of metals/metalloids in plants and enhancing crop yields.

#### Selenium (Se)

Supplementation with Se reduced the toxicity of As in rice plants by eliminating As-induced nutrient deficiency and minimizing As accumulation. A notable increase in phenolic compounds in rice roots and shoots demonstrated their potential protective function as ROS quenchers. In addition, the phenolic content and thiol metabolism-related enzymes increased with the addition of Se. The interaction between As and Se resulted in a reduction in As-induced lipid peroxidation by significantly increasing gallic acid, protocatechuic acid, and ferulic acid in the roots and increasing the levels of all other phenolic compounds in the shoots (Chauhan et al. 2017). Se reduces the oxidative damage caused by Cd and mitigates its detrimental effects on growth. Moreover, Se decreased POD and SOD activity but enhanced CAT, APX, and glutathione reductase (GR) activity (Saidi et al. 2014). Se is essential for normal functioning in both plants and the human body. Seed priming with Se is an inexpensive, safe, and environmentally friendly method for decreasing the accumulation of metalloids in the edible portions of crops and concurrently meeting the daily Se intake needs of humans (Feng et al. 2021). The addition of Se to Sb-stressed rice plants restored enzyme activity (Feng et al. 2016). The foliar application of combined sols of Se and Si to rice plants grown in contaminated soil decreased the Pb and Cd levels in the leaves and grains. Increasing sols downregulated the transcription of Cd transporter-related genes, such as *OsLCT1*, *OsHMA2*, *OsCCX2*, and *OsPCR1*, in leaves and *OsLCT1*, *OsCCX2*, *TaCNR2*, and *OSPCR1* in peduncles. In addition, sol improved leaf photosynthesis by increasing chlorophyll and ribulose 1,5-bisphosphate (RuBP) carboxylase activity levels. These sols inhibited ROS production via nicotinamide adenine dinucleotide phosphate (NADPH) oxidases and activated glutathione peroxidase, which reduced oxidative stress and damage (Wang et al. 2020).

#### Other elements

Sulfur (S) fertilization enhances rice growth and reduces Cu mobility in Cu-contaminated paddy soils by forming Fe plaques on the root surface and Cu adsorption on the root surface (Sun et al. 2016). Fe supplementation in rice plants under As stress induces increased protein and chlorophyll contents and germination capacity and increases nutritional levels (Panthri and Gupta 2019). Through an effective reduction in the rate of chromosomal aberration and an increase in the mitotic index of hairy root tip cells, calcium chloride supplementation can mitigate the growth inhibition or toxicity of Cd (Shi et al. 2014). SNP increased biomass, photosynthesis-related characteristics, and plant growth. Mn alleviated Ni-induced stress in maize seedlings by enhancing enzymatic and nonenzymatic (phenol and flavonoids) and antioxidant defense systems and decreasing the levels of oxidative stress markers (MDA and H_2_O_2_). The findings showed that SNP treatment increased the amount of organic osmolytes, glycine, and betaine and the activity of GST along with ATP and the ionic exchange capacity. The reduced production of organic acids also helps to protect maize seedlings from oxidative damage caused by Ni. SNP application increased the expression of genes linked to defense and detoxification (Abbas et al. 2023). Exogenous zinc lysine (Zn-Lys) supplementation may lessen the harmful effects of Cr and aid in plant recovery from oxidative stress by improving morphological characteristics and antioxidant enzyme activity (Zaheer et al. 2020). The addition of Zn-Lys can alleviate the toxic effects of Cd by enhancing nutritional availability, photosynthetic activity, and plant metabolites, with selective limitations on H_2_O_2_ and MDA accumulation. When used as a seed priming agent, Zn-Lys significantly reduces oxidative stress levels by enhancing the activity of nonenzymatic and enzymatic antioxidants and reducing plant metal absorption and transport through metal chelation (Hussaan et al. 2023). Mo and H_2_S play synergistic roles against AsV toxicity in bean plants by improving nitrogen metabolism and ROS, as well as chloroplast biosynthesis (Siddiqui et al. 2021).

## Phytohormone supplementation

Plant growth regulators, also known as phytohormones, are substances that, when present in very small amounts, control a variety of cellular functions as well as a plant’s reactions to environmental changes. Cytokinins (CK), auxins (Aux), gibberellins (GA), abscisic acid (ABA), ethylene (ET), salicylic acid (SA), and jasmonates (JA) are the most investigated plant hormones (Ciura and Kruk 2018). During HM exposure, phytohormones interact with redox signals and serve as signaling molecules, causing genes linked to defense mechanisms in plants to be expressed by themselves. By overexpressing genes involved in auxin biosynthesis, the exogenous administration of auxins and their precursors may improve rice resistance to HM toxicity. In addition to enhancing nutrient uptake, exogenous cytokinin administration can mitigate the negative developmental effects of HMs (Bilal et al. 2023). To combat HM toxicity, SA and its phenolic derivatives, such as methyl salicylate, can enhance the antioxidative defense system through growth regulation, facilitation of intracellular and intercellular communication, and stimulation of hormone and enzyme activity (Sharma et al. 2020). Foliar application of SA mitigates the negative effects of As stress on plant photosynthesis and growth potential through enhanced enzymatic and nonenzymatic oxidative defense systems. It minimizes oxidative stress by reducing plant ROS accumulation, and increased proline metabolism helps maintain enzyme structure and osmotic stress. This process increased the concentrations of PCs and nonprotein thiols, which reduced As uptake and translocation via metal sequestration. An increased SA supply increases carbohydrate metabolism, which reduces As-induced stress by increasing the activity of Rubisco and photosynthesis (Bano et al. 2022). The addition of melatonin (MT) increased the levels of enzymatic antioxidants and directly scavenged ROS to reduce elevated ROS levels. The incorporation of potent antioxidants such as alkaloids, proline, phenols, flavonoids, and tannins mitigates the oxidative stress caused by Ni and aids in the recovery of osmotic imbalance. A foliar spray of MT reduces Ni toxicity in fenugreek plants by decreasing its absorption and translocation in roots for effective Ni efflux from roots. It also improves photosynthetic efficiency and restores stomatal dimensions (Parwez et al. 2023). Exogenous MT could improve the production of ornamental tuberose cut flowers in HM-polluted soils by promoting an antioxidant system (Zulfiqar et al. 2023a, b). The simultaneous addition of MT and SA to sword lilies under As stress elevated their proline and protein contents, which improved their resistance (Zulfiqar et al. 2023a, b). The improved As tolerance of many plants is mainly due to the accumulation of anthocyanins, a type of flavonoid with the ability to scavenge ROS. Like GSH and PCs, anthocyanins are conjugated with As and undergo vacuolar sequestration during detoxification. Moreover, plant growth and productivity are enhanced by the application of anthocyanins (Ahammed and Yang 2022). Salicylic acid supplementation reduced the transcription level of OsCd1, which controls root Cd absorption. Increased expression of heavy metal ATPase of *Oryza sativa* (*OsHMA3*), cation/Ca exchanger 2, and pleiotropic drug resistance 9 help remove Cd from cells and transport it into vacuoles, thus reducing Cd accumulation. Decreased hemicellulose in rice cell walls induces less Cd absorption and downregulates the expression of genes responsible for Cd transport and translocation, thus mitigating Cd toxicity (Chen et al. 2023). The development of broccoli seedlings through seed priming with GSH plays a crucial role in minimizing metal toxicity in plants via metal chelation. The administration of GSH has numerous positive effects, including increasing photosynthesis and chlorophyll levels, as well as improving growth and gas exchange parameters. Thus, this technique can be employed to decrease metal uptake and increase plant resistance to lead poisoning while increasing nutrient intake (Ahmad et al. 2023).

Although monocots and dicots have morphological and physiological differences, phytohormone application provides significant benefits to both groups of plants. Monocots tend to exhibit improved root-based mechanisms, while dicots often show increased accumulation of protective compounds like anthocyanins in response to phytohormone application. Hence, phytohormones help to mitigate plant metal toxicity by increasing plant defense mechanisms and making them valuable strategies for sustainable agricultural practices in metal-contaminated environments.

## Nanoparticles (NPs)

NPs have been identified as adequate substitutes for current treatment techniques for environmental remediation caused by human activity. By foliar application of silicon nanoparticles (SiNPs), rice plants can have longer roots and shoots, a greater fresh weight, and an improved dry weight under B toxicity conditions. Treatment with SiNPs accelerated metalloid deposition in the leaf cell wall and reduced B translocation to leaves (Riaz et al. 2022). The adverse effects of Pb on coriander plants were mitigated by foliar application of SiNPs which reduced the Pb concentration in plants and enhanced the plant defense system. SiNPs reduce MDA accumulation in plant tissues and modify POD, CAT, and SOD activity in plants exposed to Pb stress (Fatemi et al. 2021). The addition of SiO_2_ to *Mentha arvensis* plants exposed to Cu increased the accumulation of nonenzymatic antioxidants such as proline and total phenols and decreased the ROS, H_2_O_2_, and O_2_ contents in the plant leaves. The maintenance of membrane integrity enhances a plant’s ability to withstand oxidative stress, leading to better yield (Aqeel et al. 2023). The absorption of Cd by plants was significantly reduced by the addition of iron oxide NPs (Wang et al. 2021a, b). The simultaneous application of titanium oxide NPs and 4-epibrassinolide significantly enhanced plant resistance to metal toxicity. The plants’ oxidative stress and ROS levels were significantly reduced upon coapplication, which improved the growth and development of the plants and increased their photosynthetic capabilities, thus aiding in minimizing HM accumulation, uptake, and translocation from the roots to the shoots (Emamverdian et al. 2022a, b). The amount and type of NPs sprayed determines the variation in plant biomass. The application of 20 mg/L nano-silica to Cd-stressed cucumber plants did not reduce Cd absorption but did increase Cd accumulation (Akhtar et al. 2020). At 10 mg/L, fullerene can significantly decrease As and Cd absorption by plants, suggesting that fullerene can slow the toxicity of metals/metalloids (Guo et al. 2019). The application of zero-valent iron nanoparticles (nZVI) increased plant biomass and reduced the amount of As that accumulated in the edible portion of *Medicago sativa*. Increased proline and NPT contents in the roots, decreased H_2_O_2_ and MDA, and reduced oxidative stress in the leaves and roots were observed in response to nZVI. When organic fertilizer is applied, it can have detrimental effects on As accumulation; thus, the combination of this fertilizer with nZVI is considered a phytostabilization approach that uses both amendments simultaneously for soils affected by As and Hg pollution (Baragaño et al. 2022). The addition of zinc oxide nanoparticles (ZnO-NPs) decreased lipoxygenase activity, electrolyte leakage, soluble protein, H_2_O_2_ content, and thiobarbituric acid reactive substances but increased antioxidant activity, proline content, glycine betaine content, tyrosine ammonia-lyase activity, phenylalanine ammonia-lyase activity, chlorophyll indices, and ultimately, plant biomass. By increasing antioxidant activity, decreasing As and Hg accumulation and translocation from roots to shoots, and modifying stomatal closure, ZnO-NPs ameliorate As and Hg toxicity in plants (Emamverdian et al. 2022a, b). When used as a soil amendment, nanobiochar reduces interchangeable Cd and enhances plant development in *Brassica chinensis* by increasing the biomass and sprouting of seeds (Liu et al. 2020). Although nanomaterials offer new agricultural tools to increase sustainable food production, the primary issue is that their release into the environment poses an urgent threat to the environment (Murali et al. 2022). At lower concentrations, nanoparticles may act as elicitors in plants; however, at higher concentrations, they can cause oxidative stress, damage plants, and cause environmental toxicity (Gowtham et al. 2024).

NPs offer viable ways to improve plant growth and lessen the negative effects of HM toxicity by increasing antioxidant activity and decreasing metal absorption when used in optimum concentrations. There are notable differences in the response of monocots and dicots to nanoparticle treatments. When treated with NPs, monocots often show improved root and shoot growth, increased photosynthetic activity, and better stress tolerance. While dicots may benefit more from improved antioxidant defense systems, they vary in terms of plant species, stressors, and the NPs used. Understanding these species-specific responses is key to optimizing nanoparticle applications for environmental remediation and agricultural enhancement.

## Soil amendments

Soil amendments include diverse materials to improve soil structure, fertility, and water retention. The toxic effects of HMs can be controlled by applying soil amendments, including polyaspartic acid, organic fertilizer, kaolinite, magnesium slag, and modified biochar/quicklime. They improve soil quality, increase the soil immobilization indices of Cd and As, and effectively reduce their deposition in edible parts (Tan et al. 2023). Reducing As and Cd toxicity in rice using iron-modified biochar (Fe-BC) is a successful strategy. Using Fe-BC instead of traditional techniques for As and Cd removal has two additional benefits: enhanced nutritional value and better-quality soil for rice crops. The absorption of Fe increases the adsorption capacity of biochar for As and Cd ions. This enhances the physiochemical properties of biochar by enhancing its permeability, functional groups, and surface area (Irshad et al. 2023). Applying charred eggshells in more significant quantities with a high calcium concentration might decrease rice yield by increasing the rhizophore pH, even if it can lower As and Cd in polluted paddy soil. This can be resolved by combining it with corncob charcoal, which improves plant growth and biomass and allows plants to absorb the required nutrients (Islam et al. 2021). The application of meta-sodium silicate (Na_2_SiO_3_) amendments reduced Pb translocation from the soil to the rice grains, decreasing the amount of Pb found in brown rice. Applying a high dose of Na_2_SiO_3_ increased PbSiO_3_ precipitation, and Pb^2+^ combined with Fe oxides in the soil reducing the bioavailability of Pb in the soil, thus decreasing Pb translocation in plant parts (Zhao et al. 2017). The application of galactoglucomannan oligosaccharides (GGMOs) increased the growth, concentration of photosynthetic pigments, photosynthetic rate, and photosystem II quantum yield of maize leaves by decreasing the concentration of Cd in the leaves of Cd-stressed plants. GGMOs are potentially biostimulant molecules that help plants increase their viability by reducing oxidative stress and membrane damage (Vivodová et al. 2023). GGMOs significantly increased the viability of protoplasts by stimulating a signaling pathway that resulted in increased biosynthesis of the maize cell wall. As a result, the cell wall contained Cd^2+^ within its structure, which reduced the amount of Cd taken up by the protoplast and increased its viability. Thus, it has potential applications in agriculture and phytoremediation (Hačkuličová et al. 2023). Crops grown on contaminated soil supplemented with lime filler or construction and demolition wastes develop similarly to those grown on noncontaminated soil and do not pose health risks when incorporated for soil remediation (Martínez-Sánchez et al. 2022).

These amendments enhance soil quality, promote metal immobilization, and reduce metal translocation to edible parts, ultimately improving plant health and productivity. Monocots, often show enhanced growth and reduced metal uptake with biochar, Si-based amendments, or phytohormone treatments, due to their different root morphology and nutrient uptake mechanisms. In contrast, dicots may respond more effectively to organic amendments and biostimulants that stimulate antioxidant systems and root growth. However, both monocots and dicots benefit from improvements in soil quality, increased microbial activity, and reduced bioavailability of toxic metals. Table 2 lists various physio-biochemical approaches to mitigate heavy metal toxicity.

**Table 2:** Physio-biochemical approaches employed to mitigate metalloid toxicity in monocot and dicot plants and elevate plant tolerance to metals

Metal/metalloid	Crop	Treatment	Mechanisms of tolerance	Reference
Inorganic supplements Monocot				
Cadmium	*Triticum aestivum*	Boron fertilizer application	Cd detoxification into vacuoles reduced the uptake of Cd in shoots at the elongation stage. Regulation of Cd detoxifying and transporter genes, increased activity of antioxidant enzymes SOD and POD	Qin et al. (2022)
Cadmium	*Oryza sativa*		Increased biomass, enhanced pectin, and lignin biosynthesis. Downregulated expression of Cd-induced transporter-related genes. Reduced Cd uptake, enhanced detoxification	Huang et al. (2021)
Arsenic	*Oryza sativa*	Iron supplementation	Phytosiderophores chelate As, thus reducing availability for uptake by the plant and vacuole sequestration by Iron plaques. Increased accumulation of macro- and micronutrients. Improved levels of germination, chlorophyll, and protein	Panthri and Gupta (2019)
Arsenic	*Oryza sativa*	Potassium humate application through growth media	Reduced ROS formation and improved root anatomical structure. Enhanced chlorophyll content, germination percentage, and reduced antioxidant stress. Minimizes bioavailability of arsenic in soil	Ray et al. (2022)
Arsenic	*Oryza sativa*	Selenium addition through hydroponics	Increased level of phenolic compounds and thiol metabolism-related enzymes. Increased uptake of nutrient elements and improved plant growth. Reduced As accumulation	Chauhan et al. (2017)
Cadmium	*Oryza sativa*		Cell wall metal adsorption and chelation, reduced metal uptake and translocation by restricted entry into protoplast. Increased antioxidant system, enhanced root length, volume, and surface area. Enhanced rice yield	Riaz et al. (2021a, b)
Zinc, Cadmium	*Oryza sativa*	Silicon fertilizer application	Decreased permeability of plasma membranes and membrane lipid peroxidation and reduced MDA contents. Increased antioxidant enzyme activities. Heavy metal sequestration inhibiting symplast metal transport	Huang et al. (2018)
Copper	*Megathyrsus maximus*		Improved biomass production, chlorophyll content, and root morphology. Reduced Cu uptake and translocation from root to shoot by induction of antioxidant system	Vieira Filho and Monteiro (2020)
Cadmium	*Triticum aestivum*	Silicon hydroponic treatment	Increased plant dry biomass, chlorophyll content, photosynthesis rate, and relative water content. Reduced ROS and MDA content. Limited uptake, accumulation, and translocation of Cd through antioxidative defense mechanism and by the formation of an apoplastic barrier	Ur Rahman et al. (2021)
Nickel	*Oryza sativa*		Increased growth and photosynthesis, biomass, and chlorophyll content. Improved water balance. Enhanced antioxidant system and glyoxalase systems, resulted in improved ROS scavenging and MGO detoxification	Hasanuzzaman et al. (2019)
Antimony	*Arundo donax*	Silicon addition through Hoagland solution	Enhanced root lignification, enhanced photosynthesis, and relative increase in biomass. Lignin deposition limits Sb uptake and translocation	Shetty et al. (2021)
Cadmium, lead	*Oryza sativa*	Silicon foliar application	Improved photosynthesis, chlorophyll in the leaves, and enhanced RuBP carboxylase activity. Reduced metal uptake through chelation	Wang et al. (2020)
Arsenic, cadmium	*Oryza sativa*	Foliar application of silica sol and iron-modified biochar soil application	Enhanced plant yield and productivity, improved soil health. Reduced levels of available As and Cd in the soils and rice grains	Pan et al. (2019)
Copper	*Oryza sativa*	Sulfur fertilization application	Increased dry biomass, enhanced growth. Metal sequestration by the formation of iron plaques, reduced Cu uptake	Sun et al. (2016)
Cadmium	*Triticum aestivum*	Zinc lysine as seed priming agent	Increased nutrient uptake, chlorophyll synthesis, and biomass accumulation. Reduced H_2_O_2_, and MDA accumulation. Reduced Cd uptake by enhanced antioxidant system	Hussaan et al. (2023)
Dicot				
Aluminum	*Pisum sativum*	Boron fertilizer application	Enhancement of rhizophore pH and stabilization of cell wall. Reduced metal uptake	Yang et al. (2022)
Aluminum	*Citrus trifoliata*	Boron supply through hydroponically	Enhanced root growth and photosynthesis. Regulated activities of oxidative enzymes. Reduced metal uptake and mobilization, restrict the entry of metal into the symplast	Riaz et al. (2018)
Arsenic	*Vicia faba*	Application of Molybdenum and hydrogen sulfide	Reduction of apoptosis, and reactive oxygen species content. Increased chlorophyll biosynthesis. Upregulated cysteine and hydrogen sulfide biosynthesis. Reduction in oxidative stress enhanced anti-oxidative system	Siddiqui et al. (2021)
Cadmium	*Helianthus annuus*	Seed priming with selenium	Enhanced the fresh weights of roots and leaves, improved activities of CAT, APX, and GR, but lowered that of SOD and POD. Increased accumulation of metal within roots not allowing it to other parts	Saidi et al. (2014)
Cadmium	*Avicennia marina*	Silicon addition	Higher amount of radial oxygen loss and Fe/Mn plaque on the roots. The apoplastic barrier in the roots and reduced Cd uptake	Zhang et al. (2013)
Aluminum	*Schinus terebinthifolius*		Improved leaf surface area, light absorption, and photosynthetic rate. Increased antioxidant system	Aguilar et al. (2023)
Chromium	*Solanum lycopersicum*		Enhanced plant growth, biomass, chlorophyll, gas exchange, water content, flavonoid content, ascorbate–glutathione cycle, and glyoxalase system. Reduced oxidative stress, proline, glycine betaine, and MGO. Reduced Cr accumulation and improved plant tolerance	Alam et al. (2021)
Cadmium, lead	*Nicotiana tabacum*	Silicon fertilizer (organic and mineral) application	Enhanced plant biomass, increased POD and APX content, and reduced MDA content. Increased photosynthesis, chlorophyll, and carotenoid content. Metal precipitation with silicon	Wang et al. (2023a, b)
Cadmium	*Solanum nigrum*	Silicon supply through Hoagland solution	Reduced H_2_O_2_ accumulation, root cell death and electrolyte leakage. Reduced Cd uptake and distribution and increased antioxidant system	Liu et al. (2013)
Arsenic	*Solanum lycopersicum*	The combined addition of silicon and sodium hydrosulfite	Increased biomass, and improved antioxidant system enzyme activities. Increased GR actively eliminates H_2_O_2_ leading to reduced oxidative stress and reduced metal uptake	Kaya and Ashraf (2022)
Copper	*Vigna radiata*	Sodium nitroprusside addition	Improved growth, photosynthesis, cell viability, and antioxidant defense, and reduced oxidative damage and cell death. Improved antioxidant defense system, reduced Cu absorption	Gaur et al. (2021)
Phytohormone application Monocot				
Arsenic	*Oryza sativa*	Methyl jasmonate and ethylene application	Increased carbohydrate metabolism and a rise in RUBISCO activity led to increased photosynthesis and induced nutrient homeostasis. Improved As-induced stress tolerance mechanism and enhanced defense system	Nazir et al. (2023)
Arsenic	*Oryza sativa*	Methyl jasmonate application	Improved growth, chlorophyll content, biomass, and photosynthesis rate. Increased antioxidant activity and the ASA–GSH cycle. Reduced As accumulation	Mousavi et al. (2020)
Arsenic	*Polianthes tuberosa*	Melatonin application	Enhanced proline and protein content. Increased growth, photosynthesis, and photosynthetic pigments. Increased antioxidant system, improves stress resistance	Zulfiqar et al. (2023a, b)
Arsenic	*Gladiolus grandiflorus*	Combined application of melatonin and salicylic acid	Increases activity of enzymatic antioxidants and enhanced protein and proline content. Improved growth, biomass, chlorophyll content, and photosynthesis	Zulfiqar et al. (2023a, b)
Cadmium	*Oryza sativa*	Exogenous addition of salicylic acid	Reduced cell wall hemicellulose level, downregulation of *OsCd1* that is responsible for root Cd absorption and enhanced production of nitric oxide in roots. Reduced metal uptake and metal sequestration in vacuoles	Chen et al. (2023)
**Dicot**				
Lead	*Brassica oleracea*	Seed priming with Glutathione	Enhanced growth, chlorophyll content, total soluble proteins, and mineral content and improved gas exchange parameters. Enhanced nutrient uptake reduces Pb stress	Ahmad et al. (2023)
Nickel	*Trigonella foenum-graecum*	Melatonin supplementation through foliar spray	Enhanced accumulation of proline, phenols, total tannins, total flavonoids, and total alkaloids concentration in plant leaves. Improved the seed trigonelline content, enhanced growth, and photosynthesis	Parwez et al. (2023)
Arsenic	*Brassica napus*	Foliar spray of salicylic acid	Enhanced growth, photosynthesis, and carbohydrate metabolism. Improved enzymatic and nonenzymatic antioxidant defense, and increased concentration of phytochelatins and non-protein thiols. Metal sequestration thus lowering As accumulation in roots and leaves	Bano et al. (2022)
Nanoparticle application Monocot				
Boron	*Oryza sativa*	Foliar application of nano-silica sol	Increased cell wall metalloid adsorption, reduced metal uptake, and enhanced antioxidant system. Increased root and shoot lengths, increased fresh weight and dry weight	Riaz et al. (2022)
Copper, cadmium	*Pleioblastus pygmaeus*	Co-application of 24-Epibrassinolide and Titanium Oxide NPs	Increased antioxidant activity and photosynthetic capacity. Reduction in amounts of MDA, H_2_O_2_, superoxide radical, soluble proteins, and percentage of electrolyte leakage. Reduced HM accumulation and translocation	Emamverdian et al. (2022a, b)
Arsenic, mercury	*Pleioblastus pygmaeus*	Zinc oxide NP application	Increased chlorophyll contents, proline content, and plant biomass. Lipoxygenase activity, electrolyte leakage, soluble protein, H_2_O_2_ content, and ROS were reduced. Increased antioxidant activity, reduced As and mercury accumulation, and translocation from roots to shoots	Emamverdian et al. (2022a, b)
Dicot				
Copper	*Mentha arvensis*	Foliar application of Si NPs	Enhanced antioxidant system. Enhanced plant growth and photosynthetic efficiency along with the activities of carbonic anhydrase and nitrate reductase	Aqeel et al. (2023)
Lead	*Coriandrum sativum*		Minimized the accumulation of MDA in plant tissues and adjusted the activities of POD, CAT, and SOD in plants. Improved plant defense system	Fatemi et al. (2021)
Cadmium, arsenic	*Cucumis sativus*	Spraying of nano-silica and fullerene	Increased Plant biomass and hormone levels, leading to increased plant growth. Metal uptake reduction	Guo et al. (2019)
Arsenic, Mercury	*Medicago sativa*	nZVI and organic fertilizer assisted phytoremediation	Diminished oxidative stress, low content of H_2_O_2_ and MDA, and the high content of proline, mainly in roots, increased NPT content. Metal/metalloid immobilization	Baragaño et al. (2022)
Soil amendments Monocot				
Cadmium, arsenic	*Oryza sativa*	Iron-modified biochar application	Increased adsorption capacity of Fe plaques to As and Cd, thus reducing metal uptake. Increased chlorophyll contents and RuBP carboxylase activities in the leaves, and enhanced photosynthesis	Irshad et al. (2023)
Lead	*Oryza sativa*	Meta sodium silicate amendment application	Precipitation of Pb-metasilicate and Pb-ferrihydrite on the root surfaces or inside the roots. Inhibition of root to stem metal translocation	Zhao et al. (2017)
Arsenic, cadmium	*Oryza sativa*	Organic fertilizer, polyaspartic acid, kaolinite, magnesium slag, and modified biochar application	Increased As accumulation in husk and grain leading to reduced accumulation in rice endosperm. Increased the soil immobilization indices of Cd and As. Promotes plant growth, improved soil quality	Tan et al. (2023)
Cadmium	*Zea mays*	Application of Galactoglucomannan oligosaccharides through hydroponics	Increased auxin synthesis, improved root growth, enhanced photosynthesis, and reduced oxidative stress. Decreased translocation of Cd from shoot to root by chelation of metal in roots Increased plant growth and cell wall biosynthesis. Improves protoplast viability and cell wall regeneration, thus reducing metal uptake and translocation	Vivodová et al. (2023) Hačkuličová et al. (2023)

*Cd* Cadmium; *SOD* Superoxide dismutase; *POD* Guaiacol peroxidase; *ROS* Reactive oxygen species; *MDA* Malondialdehyde, *Cu* Copper; *MGO* Methylglyoxal; *RuBP* Ribulose 1,5-bisphosphate; *GST* Glutathione S-transferase; *APX* Ascorbate peroxidase; *CAT* Catalase; *H*_*2*_*O*_*2*_ Hydrogen peroxide; *GSH* Glutathione; *Pb* Lead; *NPs* Nanoparticles; *Cr* Chromium; *HM* Heavy metal; *ASA* GSH–Ascorbate–glutathione; *nZVI* Zero valent iron nanoparticles; *Fe* Iron; *Sb* Antimony)

## Bioremediation

Bioremediation involves the use of microorganisms, such as bacteria, fungi, and algae, to reduce or eliminate the harmful effects of metals/metalloids under HM-stressed conditions. It is a green, effective, and economical method for degrading HMs and metalloids in soil (Ojha et al. 2023). Plant growth-promoting bacteria (PGPB), rhizobia, and arbuscular mycorrhizal fungi (AMF) are beneficial microorganisms that aid in plant uptake, sequestration, and detoxification of metals. As a natural solution, the use of AMF and the addition of NO as a signaling molecule aided in enhancing nutrient uptake, improving antioxidant enzymes, and activating the GSH and PC genes. This process aids in metal sequestration within the vacuole, thus reducing the translocation of HMs to other plant parts (Zare et al. 2023).

In addition, the use of fungi and bacteria that help plants increase resistance to HMs can be a better strategy for bioremediation strategies that are very important from an ecological and economic standpoint (Soto et al. 2019). In rice plants inoculated with As-tolerant bacterial strains, plant growth increases, and As uptake is suppressed. In addition, the bacterium-inoculated plants presented decreased total GSH and GR activity, which indicates that the bacteria help mitigate oxidative stress in plants. These strains also improved the biochemical and physiological state of plants under As stress (Singh et al. 2016a, b). Low levels of AsV have detrimental effects on the redox state of peanut plants, which can be mitigated by symbiosis with *Bradyrhizobium* sp. strains (Peralta et al. 2020). The synergistic approach of Si with two AMF species (*Claroideoglomus etunicatum* and *Rhizoglomus intraradices*) aids in modulating soil properties and improving the growth and productivity of plants in stressed soils. Increasing the activity of phosphatases and glomalin in the soil reduces the metal availability for plant uptake (Bhalla and Garg 2021). In *C. etunicatum* and *Funneliformis mosseae*, plant biomass was greatly enhanced by mycorrhizal inoculation, which also resulted in a decrease in shoot B concentrations. Thus, AMF could aid in the phytoremediation of B-contaminated soils under drought and salt stress (Liu et al. 2018). The cyanobacterial species *Spirulina platensis* is a biofertilizer that can also be used as a bioremediation tool to remove Pb, Cr, and Cd from the soil. It mainly acts by reducing membrane lipid peroxidation and Cd and Pb absorption and accumulation and thus inhibits ROS production and accumulation by modifying the antioxidant defense system (Gharib and Ahmed 2023). PGPB boosts crop yield, enabling plants to prosper in contaminated environments. For this reason, developing customized biofertilizers is crucial. Synthetic bacterial communities (SynComs) have positive effects on host plants, including increased physiology, increased plant growth, and the accumulation of proline, glutamate, and organic acids such as citrate and malate as a defense mechanism against metal stress (Flores-Duarte et al. 2023). *Pseudomonas fluorescens* and *Trichoderma* sp. effectively prevent Cd toxicity in chickpea species by producing a wide range of growth-regulating active biomolecules. Owing to their remarkable capacity for metal tolerance and other unique growth-promoting characteristics, Cd-resistant *P. fluorescens* and *Trichoderma* sp. could function as potent microbial inoculants that could be used in a simple, economical, and environmentally friendly manner to remediate HM-contaminated soils (Syed et al. 2023).

The adverse effects of As toxicity can be mitigated through the application of the plant growth-promoting bacterium *Providencia vermicola* and iron oxide NPs. This intervention not only reduced As levels in plants but also alleviated ROS, causing necessary changes in the plant ultrastructure and organic acid efflux, and strengthened the plant antioxidant system and vital nutrients (Sun et al. 2022). The coapplication of CuO NPs and *Bacillus* sp. significantly increased all the plant growth parameters under Cr stress in spinach plants. Similarly, elevated concentrations of catalase, SOD, MDA, and H_2_O_2_ were observed. In addition, total soluble protein, free amino acids, and phenolics significantly benefit from this coapplication (Ali et al. 2023). The metal removal process utilizing a copper iodide nanocomposite with acidophilic bacterial cocultures was reasonably effective at removing Cr and Zn (Akhtar et al. 2020).

Biochar is a carbon-enriched substance synthesized by the pyrolysis of plant materials and is available from agricultural byproducts (Nzediegwu et al. 2019). Biochar can lower soil metal availability through ionic exchange, surface ligand-mediated binding of metal ions, and electrostatic interactions (Liu et al. 2020). Biochar reduces bioavailable Cu, increases plant shoot and root growth and decreases soil metal toxicity in plants (Tang et al. 2023). The significant characteristics of biochar, such as its large internal surface area, negative charge, and resistance to degradation, make it an excellent remediation material for contaminated soils. This bioremediation strategy is of utmost importance because it not only addresses soil pollution and enhances soil quality but also reduces the worldwide environmental challenge of solid waste management due to its cost-effectiveness (Wang et al. 2021a, b; Raza et al. 2022). Table 3 illustrates how interactions between microbes and plants through symbiotic relationships are crucial for increasing plant tolerance to metals. Furthermore, using plants for metal phytoextraction and soil remediation is a sustainable solution. Bioremediation offers enhanced growth and HM tolerance. However, only a few studies have compared monocots and dicots for a single treatment.

**Table 3:** Biological approaches in improving plant tolerance to metals and metalloid toxicity in monocot and dicot plants

Metal/metalloid	Crop	Treatment	Mechanism of tolerance	Reference
Monocot				
Arsenic	*Oryza sativa*	As-tolerant bacterial inoculum application	Enhanced plant growth, reduced activity of total GSH and GR. Reduced As uptake	Singh et al. (2016a, b)
Arsenic	*Triticum aestivum*	As-resistant and plant growth promoter microorganisms inoculation	Relative expression of plant metallothionein, SOD, APX, and PC synthase genes were overexpressed leading to higher antioxidant response system	Soto et al. (2019)
Boron	*Puccinellia tenuiflora*	Mycorrhizal inoculation	Increased plant biomass, and improved phosphorus and potassium content. Reduced B translocation	Liu et al. (2018)
Cadmium	*Zea mays*	Application of sodium nitroprusside and AMF	Stimulate intracellular detoxification, Phytochelation, and metal sequestration within vacuole resulting in reduced metal uptake and translocation	Zare et al. (2023)
Cadmium, Arsenic	*Oryza sativa*	Biochar application	Sequestration of As and Cd leading to reduced metalloid uptake with enhanced biomass, and improved growth	Islam et al. (2021)
Dicot				
Arsenic	*Cajanus cajan*	Exogenous addition of silicon and AMF	Enhanced soil glomalin and phosphatase activity, increased plant nutrient acquisition, biomass, and chlorophylls; it boosted the activities of starch hydrolytic enzymes increase in total soluble sugars. Decreased metal availability in soil	Bhalla and Garg (2021)
Arsenic	*Arachis hypogaea*	Plant inoculation with *Bradyrhizobium* sp. strains	Increased chlorophyll and carotene content. Enhanced growth and modulation of redox response. Reduced translocation of As to edible parts	Peralta et al. (2020)
Arsenic	*Trachyspermum ammi*	Combined application of plant growth-promoting bacteria and iron oxide NPs	Improved plant growth and composition and balanced exudation of organic acids. Decreased As concentration in the plant tissues	Sun et al. (2022)
Chromium	*Spinacia oleracea*	Co-application of copper NPs and metal-tolerant *Bacillus* sp	Elevated concentrations of CAT, SOD, MDA, H_2_O_2_, total soluble protein, free amino acids, and total phenolics were observed. Increased antioxidant system, reduced metal absorption	Ali et al. (2023)

*GSH* Glutathione; *GR* Glutathione reductase; *SOD* Superoxide dismutase; *APX* Ascorbate peroxidase; *PC* Phytochelatin; *B* Boron; *AMF* Arbuscular mycorrhizal fungi; *As* Arsenic; *Cd* Cadmium; *MDA* Malondialdehyde; *CAT* Catalase; *POD* Guaiacol peroxidase; *H*_*2*_*O*_*2*_ Hydrogen peroxide, *NPs* Nanoparticles

## Phytoremediation

Phytoremediation is an affordable, environmentally friendly, and ecologically sustainable technique (Khan et al. 2023). The use of plants to remove, adsorb, detoxify, and hyperaccumulate harmful pollutants from soil, water, and air is referred to as phytoremediation, which is a crucial method for detoxifying the environment and minimizing the risk of potential cancer-causing substances and other harmful toxins to humans and animals (Cobbett and Meagher 2002). Phytoextraction is an economical method for removing metals from these soils via the use of plants that hyperaccumulate metals by transferring and concentrating the metals from the soil to plant parts. The use of plants to decrease the bioavailability of contaminants in the environment is known as phytostabilization (Garbisu and Alkorta 2001). Phytoremediation, a more effective means of restoring sites contaminated with metals and metalloids, has drawn much attention recently (Mendoza-Cózatl et al. 2011). Fast-growing plant species with unique genes found in metal-hyperaccumulating plants and microbes that can tolerate, build up, and purify metals and metalloids can be effectively used in phytoremediation (LeDuc and Terry 2005). Although spinach plants have a high HM content in their tissues, there are no obvious symptoms of toxicity, suggesting that spinach plants are good candidates for phytoextraction. However, in plants grown for phytoremediation, the Cd and Pb concentrations in edible parts exceed the safe limits set by the US EPA, raising concerns about accidental food chain exposure (Chaturvedi et al. 2019). The hyperaccumulator nature of *Brassica juncea* enables it to absorb large amounts of metalloids without transferring them to the shoots. It plays a crucial role in metal retention to prevent harmful buildup in the shoots (Ahmad et al. 2020). Since As is found exclusively in root systems and has not been found to have translocated into the shoots, *Brassica juncea* can be utilized as a practical and economically feasible phytoaccumulator in As-contaminated areas (Rahman et al. 2016). By decreasing the uptake and accumulation of As/Cd, decreasing the H_2_O_2_ content, and increasing the levels of protective compounds such as antioxidant enzymes, Si supplementation reduces the toxicity of As/Cd and restores the normal growth and development of the hyperaccumulator *Isatis cappadocica*. Si application cannot contribute to increased phytoextraction, but it can increase the phytostabilization of plants at sites contaminated with As and Cd (Azam et al. 2021). Anthocyanin-enriched hyperaccumulator plants can be used for the phytoremediation of As-polluted soils (Ahammed and Yang 2022).

Due to its capacity to store HMs in its roots, *Hordeum vulgare* appears to be a good phytostabilizing plant (Dago et al. 2014). AMF mitigates Zn phytotoxicity primarily through binding trace metals via metal adsorption, facilitating the ability of plants to effectively limit both the uptake of metals from soils and their subsequent translocation to plant shoots. In many moderately trace metal-polluted sites, AMF aid plants in thriving and utilizing phytostabilization through improved P uptake and balanced mineral nutrition. It is thus used as a phytoremediation strategy (Christie et al. 2004).

Phytoextraction involves the use of plants to extract toxins from water and soil. Since castor bean plants are somewhat resistant to As, they can be effectively used to revegetate areas contaminated with As (Melo et al. 2009). The application of rhizobial strain inoculum to horse gram plants aids in the phytoextraction of Co from contaminated soils (Edulamudi et al. 2023). As a hyperaccumulator, *Pteris vittata* has a more effective defense system that offers sufficient biochemical mechanisms to withstand stress and allow its biomass to hyperaccumulate. As a result, it can be applied in phytoremediation to facilitate phytoextraction (Tiwari and Sarangi 2017). The combination of *Hibiscus rosa*-*sinensis* and *Acidithiobacillus thiooxidans* could be used as an excellent hyperaccumulator for extracting metal pollution from polluted soil because preisolated *A. thiooxidans* showed remarkable multimetal tolerance up to 800 μg mL^−1^ Cr, Cd, Pb, and Mn (Thanh et al. 2023). Although *Dahlia pinnata* is a weak candidate for phytoextraction due to its nonaccumulator nature, which primarily accumulates As in roots and has limited As aerial translocation, its propensity to retain the majority of absorbed As in its roots makes it a viable option for phytostabilization at As-contaminated sites (Raza et al. 2019). HM pollution substantially decreased the amount of ALA-D activity in the leaves of onion plants in metal-contaminated areas, suggesting that this plant is a valuable tool for detecting HM contamination in soil. To assess the level of Pb pollution in the environment, ALA-D activity can be utilized as an extremely sensitive biomarker (Gashi et al. 2020). The utilization of the inherent capacities of plants that hyperaccumulate metals may effectively address various issues associated with metal toxicity.

## Omics approaches

Integrating multiomics data facilitates the identification of key genes and pathways involved in metal tolerance, enabling targeted molecular interventions for crop improvement. Research in the fields of phenomics, proteomics, ionomics, metabolomics, miRNAomics, and genomics has progressed in recent years, making it feasible to develop plants that are tolerant to toxic metals/metalloids and to identify the molecular regulators associated with tolerance (Singh et al. 2016a, b; Raza et al. 2022; Shi et al. 2024). The responses of soil microbes and plants to HMs have been extensively studied by applying genomic, proteomic, and metabolomic techniques (Gebhardt et al. 2005; Uchimiya et al. 2020). The identification of probable genes involved in HM tolerance through transcriptome analysis, such as RNA sequencing (RNA-Seq), can be applied to transform nonhyperaccumulator plants into suitable candidates for phytoremediation. Metallomics and metallophenolomics are integrated methods for examining the interactions of metals or metalloids with plant phenolics (Fedenko et al. 2022). Comparative analysis via the microarray technique aids in the recognition of putative gene sets exhibiting differentially regulated genes in response to HMs and offers new perspectives on tolerance and defense mechanisms in plants against HM stress (Di Baccio et al. 2011).

Through transcriptome analysis, genes that were differentially expressed in HM-exposed plants can be identified, and after validation through qRT‒PCR analysis, this approach can be implemented successfully to create transgenic plants. A total of 127,977 unigenes in barley exposed to excessive boron and 10 differentially expressed genes were validated (Tombuloglu et al. 2015). Five essential metabolic pathways involving 89 differentially expressed metabolites were identified via metabolic analysis under Cr stress. The associations and stress response techniques between soil bacterial populations and *Iris tectorum* plants under Cr stress were revealed using a multiomics approach, which provided a theoretical framework for bioremediation (Sheng et al. 2023). Sb(III) and Sb(V) exposure significantly influences the metabolite profiles of the rice plants, altering the levels of metabolites involved in carbon metabolism, amino acid metabolism, and photosynthesis (Zhu et al. 2022). Whole-transcriptome RNA-seq analysis of *Brassica napus* revealed that the addition of sewage sludge to soil caused a significant increase in the content of HMs. In response to sewage sludge supplementation, 555 DEGs related to photosynthesis, carbohydrate metabolism, photosystem repair, and HMs were detected; these genes were among the top significantly enriched GO terms. A deeper understanding of any approach to enhancing metal/metalloid tolerance is possible via transcriptomic approaches (Jaskulak et al. 2022). According to transcriptome and metabolomic studies, Pb mainly alters the contents of flavonoids, organic acids, amino acids, and carbohydrates and influences carbon, glycine and dicarboxylic acid metabolism, and amino acid biosynthesis pathways (Meng et al. 2022).

Integrating multiomics data offers a powerful approach for identifying the key genes, pathways, and mechanisms involved in metal and metalloid tolerance. Through transcriptome analysis and metabolic profiling, researchers can identify genes and metabolites that are differentially expressed in response to HM exposure, leading to the development of transgenic plants with enhanced tolerance. Ultimately, the use of omics technologies holds great potential for optimizing metal tolerance in both monocot and dicot plants, although species-specific differences must be considered when tailoring interventions effectively for each plant group. Hence, omics techniques help define the root cause of this problem by identifying appropriate genes and can be very effective in creating solutions to problems related to metalloid and HM stress through the subsequent modification of genes and pathways, leading to improved metal tolerance.

### Molecular approach

One promising phytoremediation method is identifying genes that are distinct from those of naturally occurring hyperaccumulators and their subsequent transfer to quickly growing species (De Souza et al. 2002). According to previous studies, the *BnPCR* (*Brassica napus Plant Cadmium Resistance)* family can transport Cd^2+^ and Cu^2+^ and has a significant role in response to HM stress. With the use of this knowledge, transgenic rapeseed that is resistant to HM can be bred, which is crucial for environmental preservation and sustainable agriculture. Transgenic plants that were grown on Cd-containing media demonstrated that overexpression of *BnPCR10.1* in Arabidopsis plants enhanced their tolerance to Cd (Liu et al. 2023). The upregulation of *phytochelatin synthase gene* of *Allium sativum* (*AsPCS1*) and *GSH1* (glutathione) can increase the tolerance and adsorption of As and Cd in transgenic *Arabidopsis thaliana*, which can be employed as a method of phytoremediation to purify water and soil contaminated by metalloids or HMs (Guo et al. 2008). Transgenic plants overexpressing the *glutathione S-transferase gene* of *Oryza sativa* (*OsGSTU40*) exhibited improved plant development and reduced As absorption into shoots. Compared with wild-type plants, transgenic plants exhibit less lipid peroxidation under As stress (Pandey et al. 2023). The ATP binding cassette transporter C-type (ABCC) phytochelatin carriers AtABCC1 and AtABCC2 facilitate As tolerance in Arabidopsis. Plants overexpressing *AtABCC1* and phytochelatin synthase *AtPCS1 genes* showed enhanced resistance to As. Thus, plants may be engineered to be more suited for phytoremediation or to have less metal buildup in edible regions through the manipulation of vacuolar phytochelatin (PC) transporters (Song et al. 2010). The greater As tolerance displayed by the *AtACR2* gene (*arsenic reductase 2*) of *Arabidopsis thaliana* in transgenic tobacco plants is the consequence of greater As(III) sequestration within the vacuole, which is the least harmful. The high germination rate of the *AtACR2 t*ransgenic seeds validates their better capacity to overcome crucial stages of growth during As stress. Compared with nontransgenic controls, transgenic tobacco plants expressing the *AtACR2* gene can accumulate more As in their roots and less in their shoots. Therefore, in contaminated soil, this technique can be successful (Nahar et al. 2017).

Bidirectional As(III) channels known as plant aquaporins, or nodulin 26-like intrinsic proteins (NIPs), permit the movement of Sb(OH)_3_ and As(OH)_3_ through membranes. NIPs are found in *A. thaliana*, *Oryza sativa*, and *Lotus japonicus* when they are cloned and inserted into the yeast expression vector pYES2.1 for the uptake of As in media. This strategy is helpful in regions where As contamination is high, and it may be used to improve food and water safety. As a phytoremediation technique, it might eliminate harmful metalloids from the environment and prevent their accumulation in crops (Bienert et al. 2008). In *Xanthomonas campestris*, oxyR, a regulator of the As inducible oxidative stress gene, plays an important role in the defense against As exposure (Sukchawalit et al. 2005). Genes involved in oxidative stress and RNA‒protein production were shown to be transcriptionally controlled in an arsenic-tolerant type 1 (ATT1) rice mutant generated through radiation-induced mutagenesis. Fifty genes exhibited variations in DNA polymorphisms in upstream regions compared with wild-type exon regions, improving the ability of the mutant to tolerate As (Hwang et al. 2017). In rice, *the ATP binding cassette transporter G (ABCG)* family encodes *Pleiotropic Drug Resistance 20* of *Oryza sativa* (*OsPDR20*), a membrane protein specific to the plasma membrane of rice plants. Since *OsPDR20* facilitates the transport of Cd ions across cellular membranes, it plays a critical role in controlling rice tolerance and accumulation of Cd. When *OsPDR20* was knocked down in rice plants, growth phenotypes were hampered, and Cd accumulation increased. The growth of RNAi plants is associated with *OsPDR20* mutation-induced Cd toxicity, highlighting the function of *OsPDR20* in preserving plant fitness in the context of metal stress (Li et al. 2024). In sorghum, *SbHKT2b* expression improved Cd tolerance and ROS scavenging and decreased Cd absorption. The transcription of *SbHKT2b* is directly regulated by the transcription factor *SbWRKY54*; thus, overexpression of the latter reduces the accumulation of ROS and Cd. The overexpression of both *SbWRKY54–SbHKT2b* is crucial for the response of sorghum to Cd stress (Wang et al. 2023a, b). Metal ion transport in plants is aided by a gene family known as natural resistance-associated macrophage protein (NRAMP). In peaches, *PpNRAMP5* suppression via virus-induced gene silencing improved Mn tolerance. By significantly decreasing the amount of Mn in roots, *PpNRAMP5* silencing effectively decreased the toxicity of Mn, improved the photosynthetic apparatus, and decreased the degradation of chlorophyll. As a result, *PpNRAMP5*-silenced plants showed reduced oxidative stress damage and decreased H_2_O_2_ contents (Noor et al. 2023).

The *lipoxygenase gene* of *Malus domestica* (*MdLOX3*) controls plant growth and development and improves various plant parameters, such as taproot length, fresh weight, chlorophyll content, anthocyanin content, MDA content, and relative electrical conductivity, under HM stress. Through improved ROS clearance, *MdLOX3* overexpression in apple and Arabidopsis plants effectively increased plant resistance to Zn stress. Since homologous mutants exhibit greater sensitivity to Zn, it can be concluded that *MdLOX3* has a beneficial effect on apples under Zn stress, thereby expanding the range of applications for *LOX3* in other plant species (Chen et al. 2024). A HM ion transporter, plant Cd resistance protein (PCR), was cloned from *Populus euphratica*. Under conditions of HM stress, the *PePCR10*-overexpressing plants presented significantly greater plant height, root length, fresh weight, and dry weight than the wild-type plants. *PePCR10* improved poplar Cd/Al tolerance and decreased the HM content in the plants. Thus, *PePCR10* represents a promising genetic resource for efficiently mitigating the accumulation of HMs in crops (Guan et al. 2023). *Pleiotropic drug resistance* (*PDR*) genes are essential for the accumulation and transport of HMs in plants. Cd tolerance was conferred by the ectopic expression of *RsPDR8*, a *PDR* from radish. Furthermore, the transient transformation of *RsPDR8* enhanced radish membrane permeability and promoted ROS scavenging, thereby positively regulating Cd tolerance. Furthermore, compared with wild-type Arabidopsis plants, *RsPDR8* overexpression led to an increase in root elongation and a decrease in the accumulation of Cd (Zhang et al. 2023a, b). Table 4 lists the molecular strategies that have been used to overcome HM and metalloid toxicity and enhance plant tolerance in monocot and dicot plants. Molecular techniques offer an effective strategy for improving plant resistance to metals.

**Table 4:** Molecular approaches to enhance plant metal tolerance in monocot and dicot plants

Metal/metalloid	Crop	Treatment	Mechanisms of tolerance	Reference
Monocot				
Arsenic	*Oryza sativa*	As-tolerant type rice mutant by gamma irradiation mutagenesis	Downregulation of genes related to stress response and the upregulation of genes involved in photosynthesis, transport, and cellular biosynthesis. DNA polymorphisms in upstream regions enhance As tolerance	Hwang et al. (2017)
Cadmium	*Sorghum bicolor*	Overexpression of *SbWRKY54* and *SbHKT2b* gene by inserting into the CaMV 35S promoter driven pCambia1301 vector	Enhanced ROS scavenging ability reduced oxidative damage, and improved membrane stability. Increased POD, SOD, and CAT activity	Wang et al. (2023a, b)
Arsenic	*Oryza sativa*	Overexpression of *OsGSTU40* under the control of the maize UBIL promoter	Reduced As translocation resulting in enhanced growth, and yield	Pandey et al. (2023)
Dicot				
Cadmium, Copper	*Arabidopsis thaliana*	Overexpression of *35 s-BnPCR10.1* lines through Agrobacterium by floral dip method	Metal transporter genes were downregulated leading to enhanced tolerance and detoxification of Cd, Cu, and reduced absorption in roots	Liu et al. (2023)
Arsenic	*Arabidopsis thaliana*	Overexpression of ABCC-type phytochelatin transporter	Higher germination of transgenic seeds and reduced root to shoot metal translocation	Song et al. (2010)
Arsenic	*Nicotiana tabacum*	Transgenic tobacco plants expressing the *AtACR2* gene of *Arabidopsis thaliana*	Enhanced arsenite sequestration into the vacuole. Increased growth, biomass, and enhanced germination	Nahar et al. (2017)
Cadmium, Arsenic	*Arabidopsis thaliana*	Overexpressing *GSH1* and *AsPCS1* derived from garlic and baker’s yeast by agrobacterium-mediated transformation	Increased tolerance to toxic metal with enhanced production of total thiol content	Guo et al. (2008)
Manganese	*Prunus persica*	*PpNRAMP5* suppression via virus-induced gene silencing	Improved manganese tolerance, by activation of antioxidant system, Increased photosynthetic rate, reduced chlorophyll degradation, and reduced oxidative stress damage and H_2_O_2_ content	Noor et al. (2023)
Zinc	*Lycopersicon esculentum*, *Malus pumila*, *Arabidopsis thaliana*	Ectopic expression of* MdLOX3*	Increased zinc tolerance improved the ability to clear ROS. Enhanced plant taproots length, plant fresh weight, chlorophyll, and anthocyanins content	Chen et al. (2024)
Cadmium, Aluminum	*Populus euphratica*	*PePCR10-*overexpression	Decreased metal accumulation. Enhanced plant height, root length, fresh weight, and dry weight	Guan et al. (2023)
Cadmium	*Raphanus sativus*	Transient transformation of *RsPDR8*	Decreased Cd accumulation due to increased ROS scavenging and enhanced membrane permeability and increased root elongation	Zhang et al. (2023a, b)

*As* Arsenic; *Cd* Cadmium; *ROS* Reactive oxygen species; *SOD* Superoxide dismutase; *CAT* catalase; *POD* Guaiacol peroxidase; *Cu* Copper; *OsGSTU40 glutathione S-transferase gene* of *Oryza sativa; BnPCR10.1*, Plant cadmium-resistance protein of *Brassica napus*; *GSH1* Glutathione; *AsPCS1 Phytochelatin synthase gene* of *Allium sativum; AtACR2 Arsenic reductase gene* of *Arabidopsis thaliana; PpNRAMP5* natural resistance-associated macrophage protein of *Prunus persica*; *MdLOX3 lipoxygenase gene* of *Malus domestica*; *PePCR10* Plant cadmium-resistance protein of *Populus euphratica; RsPDR8 Pleiotropic drug resistance gene* of *Raphanus sativus*)

In conclusion, genetic engineering plays a key role in the development of plants with enhanced resistance to HMs and metalloids by manipulating genes involved in metal transport, detoxification, and stress response and thus increasing the suitability of these plants for phytoremediation. Transgenic monocots often show more pronounced resistance due to their faster growth rates and specific mechanisms of metal uptake and detoxification. In contrast, dicots benefit from genetic modifications in genes related to oxidative stress and metal sequestration, which improves growth under metal stress. Molecular strategies provide promising tools for improving plant resilience, but understanding species-specific responses is essential for optimizing their use in phytoremediation efforts.

## Conclusion

Plants combat heavy metal stress through marginalization, chelation, compartmentalization, efflux pumps, and improved antioxidant defense mechanisms. Plant metal tolerance via various methods, such as inorganic supplements, phytohormones, and soil amendments, as well as bioremediation techniques, such as phytoremediation and phytoextraction are extensively used. Currently, novel approaches such as developing transgenic plants, omics approaches, and nanomaterial applications to mitigate metal/metalloid toxicity are crucial. This review focuses primarily on documenting several physio-biochemical, biological, and molecular strategies to increase metal and metalloid tolerance in plants. We also compared these mitigative approaches in monocot and dicot plants. Although the effects of metals and metalloids on plants are available in the literature, in-depth analyses of sequestration, transport, and detoxification mechanisms need to be performed systematically. Future studies investigating the enhancement of plant heavy metal tolerance through a comparative analysis of monocot and dicot species may provide clearer insights into the underlying mechanisms involved. In addition, more research on the implementation of molecular strategies to enhance plant HM tolerance needs to be done systematically in both monocot and dicot plants. Simultaneous physiological and transcriptomic analysis of both monocot and dicot may reveal the mechanisms required for successful detoxification and further their effective use in phytoremediation. A thorough understanding of the mechanisms underlying different approaches is essential for effectively reducing HM toxicity in plants.

## Data Availability

Data sharing is not applicable to this article as no new data were created in this study.
